# Supplementary material for: Nuclear High Mobility Group A2 (HMGA2) Interactome Revealed by Biotin Proximity Labeling
Source: Int J Mol Sci. 2023 Feb 20;24(4):4246. doi: 10.3390/ijms24044246 (PMC9966875; doi:10.3390/ijms24044246)
Supplement: Supplementary file 1 [file ijms-24-04246-s001.zip › ijms-2219670-supplementary.pdf]

ID	Term	Ontology Source	Term PValue	Term PValue Corrected with Benjamini-Hochberg	Group PValue	Group PValue Corrected with Benjamini-Hochberg	GOGroups	% Associated Genes	Nr. Genes	Associated Genes Found
R-HSA:4615885	SUMOylation of DNA replication proteins	REACTOME_Pathways_25.05.2022	8.31E-04	8.55E-04	8.31E-04	8.31E-04	Group 0	6.67	3.00	[CDCA8, TOP2A, TOP2B]
R-HSA:162587	HIV Life Cycle	REACTOME_Pathways_25.05.2022	3.12E-05	5.21E-05	5.11E-06	7.15E-06	Group 1	4.03	6.00	[GTF2B, PSIP1, SSRP1, SUPT16H, XRCC5, XRCC6]
R-HSA:167172	Transcription of the HIV genome	REACTOME_Pathways_25.05.2022	2.98E-03	2.98E-03	5.11E-06	7.15E-06	Group 1	4.29	3.00	[GTF2B, SSRP1, SUPT16H]
R-HSA:6796648	TP53 Regulates Transcription of DNA Repair Genes	REACTOME_Pathways_25.05.2022	2.11E-03	2.14E-03	5.11E-06	7.15E-06	Group 1	4.84	3.00	[MDC1, SSRP1, SUPT16H]
R-HSA:162587	HIV Life Cycle	REACTOME_Pathways_25.05.2022	3.12E-05	5.21E-05	3.12E-05	3.64E-05	Group 2	4.03	6.00	[GTF2B, PSIP1, SSRP1, SUPT16H, XRCC5, XRCC6]
R-HSA:162592	Integration of provirus	REACTOME_Pathways_25.05.2022	5.46E-06	1.47E-05	3.12E-05	3.64E-05	Group 2	33.33	3.00	[PSIP1, XRCC5, XRCC6]
R-HSA:162594	Early Phase of HIV Life Cycle	REACTOME_Pathways_25.05.2022	2.33E-05	4.29E-05	3.12E-05	3.64E-05	Group 2	21.43	3.00	[PSIP1, XRCC5, XRCC6]
R-HSA:164843	2-LTR circle formation	REACTOME_Pathways_25.05.2022	2.29E-06	6.67E-06	3.12E-05	3.64E-05	Group 2	42.86	3.00	[PSIP1, XRCC5, XRCC6]
R-HSA:167172	Transcription of the HIV genome	REACTOME_Pathways_25.05.2022	2.98E-03	2.98E-03	3.12E-05	3.64E-05	Group 2	4.29	3.00	[GTF2B, SSRP1, SUPT16H]
R-HSA:157579	Telomere Maintenance	REACTOME_Pathways_25.05.2022	6.43E-06	1.50E-05	9.65E-12	1.69E-11	Group 3	5.31	6.00	[ATRX, H2AC14, H2AC6, H2BC13, H4-16, RFC1]
R-HSA:5693532	DNA Double-Strand Break Repair	REACTOME_Pathways_25.05.2022	1.18E-09	8.24E-08	9.65E-12	1.69E-11	Group 3	5.95	10.00	[BAZ1B, H2BC13, H4-16, LIG3, MDC1, PARP1, RFC1, RIF1, XRCC5, XRCC6]
R-HSA:5693538	Homology Directed Repair	REACTOME_Pathways_25.05.2022	2.02E-05	3.83E-05	9.65E-12	1.69E-11	Group 3	4.35	6.00	[H2BC13, H4-16, LIG3, MDC1, PARP1, RFC1]
R-HSA:5693565	Recruitment and ATM-mediated phosphorylation of repair and signaling proteins at DNA double strand breaks	REACTOME_Pathways_25.05.2022	2.77E-04	3.03E-04	9.65E-12	1.69E-11	Group 3	5.19	4.00	[BAZ1B, H2BC13, H4-16, MDC1]
R-HSA:5693571	Nonhomologous End-Joining (NHEJ)	REACTOME_Pathways_25.05.2022	3.20E-07	1.49E-06	9.65E-12	1.69E-11	Group 3	8.82	6.00	[H2BC13, H4-16, MDC1, RIF1, XRCC5, XRCC6]
R-HSA:5693606	DNA Double Strand Break Response	REACTOME_Pathways_25.05.2022	2.91E-04	3.14E-04	9.65E-12	1.69E-11	Group 3	5.13	4.00	[BAZ1B, H2BC13, H4-16, MDC1]
R-HSA:73884	Base Excision Repair	REACTOME_Pathways_25.05.2022	8.56E-08	5.99E-07	9.65E-12	1.69E-11	Group 3	7.61	7.00	[H2AC14, H2AC6, H2BC13, H4-16, LIG3, PARP1, RFC1]
R-HSA:73933	Resolution of Abasic Sites (AP sites)	REACTOME_Pathways_25.05.2022	5.44E-04	5.69E-04	9.65E-12	1.69E-11	Group 3	7.69	3.00	[LIG3, PARP1, RFC1]

S1a

ID	Term	Ontology Source	Term PValue	Term PValue Corrected with Benjamini-Hochberg	Group PValue	Group PValue Corrected with Benjamini-Hochberg	GOGroups	% Associated Genes	Nr. Genes	Associated Genes Found
R-HSA:1221632	Meiotic synapsis	REACTOME_Pathways_25.05.2022	2.95E-08	3.44E-07	3.94E-12	1.38E-11	Group 4	8.86	7.00	[H2AC14, H2AC6, H2BC13, H4-16, RAD21, SMC3, STAG2]
R-HSA:1474165	Reproduction	REACTOME_Pathways_25.05.2022	1.04E-07	6.65E-07	3.94E-12	1.38E-11	Group 4	5.59	8.00	[H2AC14, H2AC6, H2BC13, H3C13, H4-16, RAD21, SMC3, STAG2]
R-HSA:1500620	Meiosis	REACTOME_Pathways_25.05.2022	2.32E-08	3.25E-07	3.94E-12	1.38E-11	Group 4	6.78	8.00	[H2AC14, H2AC6, H2BC13, H3C13, H4-16, RAD21, SMC3, STAG2]
R-HSA:2468052	Establishment of Sister Chromatid Cohesion	REACTOME_Pathways_25.05.2022	8.24E-08	6.41E-07	3.94E-12	1.38E-11	Group 4	36.36	4.00	[PDS5A, RAD21, SMC3, STAG2]
R-HSA:2470946	Cohesin Loading onto Chromatin	REACTOME_Pathways_25.05.2022	5.26E-08	4.60E-07	3.94E-12	1.38E-11	Group 4	40.00	4.00	[PDS5A, RAD21, SMC3, STAG2]
R-HSA:2500257	Resolution of Sister Chromatid Cohesion	REACTOME_Pathways_25.05.2022	7.43E-07	2.89E-06	3.94E-12	1.38E-11	Group 4	5.56	7.00	[AHCTF1, CDCA8, PDS5A, RAD21, RCC2, SMC3, STAG2]
R-HSA:2990846	SUMOylation	REACTOME_Pathways_25.05.2022	3.52E-09	6.16E-08	3.94E-12	1.38E-11	Group 4	5.32	10.00	[CBX5, CDCA8, H4-16, MDC1, PARP1, RAD21, SMC3, STAG2, TOP2A, TOP2B]
R-HSA:3108214	SUMOylation of DNA damage responseand repair proteins	REACTOME_Pathways_25.05.2022	1.52E-05	2.96E-05	3.94E-12	1.38E-11	Group 4	6.49	5.00	[MDC1, PARP1, RAD21, SMC3, STAG2]
R-HSA:3108232	SUMO E3 ligases SUMOylate target proteins	REACTOME_Pathways_25.05.2022	2.57E-09	6.00E-08	3.94E-12	1.38E-11	Group 4	5.49	10.00	[CBX5, CDCA8, H4-16, MDC1, PARP1, RAD21, SMC3, STAG2, TOP2A, TOP2B]
R-HSA:68884	Mitotic Telophase/Cytokinesis	REACTOME_Pathways_25.05.2022	1.78E-07	9.56E-07	3.94E-12	1.38E-11	Group 4	30.77	4.00	[PDS5A, RAD21, SMC3, STAG2]
R-HSA:9018519	Estrogen-dependent gene expression	REACTOME_Pathways_25.05.2022	1.51E-07	8.83E-07	3.94E-12	1.38E-11	Group 4	5.33	8.00	[H2AC14, H2AC6, H2BC13, H3C13, H4-16, RAD21, SMC3, STAG2]

S1b

ID	Term	Ontology Source	Term PValue	Term PValue Corrected with Benjamini-Hochberg	Group PValue	Group PValue Corrected with Benjamini-Hochberg	GOGroups	% Associated Genes	Nr. Genes	Associated Genes Found
R-HSA:110328	Recognition and association of DNA glycosylase with site containing an affected pyrimidine	REACTOME_Pathways_25.05.2022	1.12E-04	1.31E-04	7.66E-12	1.79E-11	Group 5	6.56	4.00	[H2AC14, H2AC6, H2BC13, H4-16]
R-HSA:110329	Cleavage of the damaged pyrimidine	REACTOME_Pathways_25.05.2022	1.12E-04	1.31E-04	7.66E-12	1.79E-11	Group 5	6.56	4.00	[H2AC14, H2AC6, H2BC13, H4-16]
R-HSA:110330	Recognition and association of DNA glycosylase with site containing an affected purine	REACTOME_Pathways_25.05.2022	8.01E-05	1.04E-04	7.66E-12	1.79E-11	Group 5	7.14	4.00	[H2AC14, H2AC6, H2BC13, H4-16]
R-HSA:110331	Cleavage of the damaged purine	REACTOME_Pathways_25.05.2022	8.01E-05	1.04E-04	7.66E-12	1.79E-11	Group 5	7.14	4.00	[H2AC14, H2AC6, H2BC13, H4-16]
R-HSA:171306	Packaging Of Telomere Ends	REACTOME_Pathways_25.05.2022	5.98E-05	8.37E-05	7.66E-12	1.79E-11	Group 5	7.69	4.00	[H2AC14, H2AC6, H2BC13, H4-16]
R-HSA:212165	Epigenetic regulation of gene expression	REACTOME_Pathways_25.05.2022	2.30E-06	6.44E-06	7.66E-12	1.79E-11	Group 5	4.70	7.00	[BAZ1B, CBX3, H2AC14, H2AC6, H2BC13, H3C13, H4-16]
R-HSA:5250913	Positive epigenetic regulation of rRNA expression	REACTOME_Pathways_25.05.2022	2.28E-07	1.14E-06	7.66E-12	1.79E-11	Group 5	6.60	7.00	[BAZ1B, CBX3, H2AC14, H2AC6, H2BC13, H3C13, H4-16]
R-HSA:5250924	B-WICH complex positively regulates rRNA expression	REACTOME_Pathways_25.05.2022	1.81E-06	5.77E-06	7.66E-12	1.79E-11	Group 5	6.59	6.00	[BAZ1B, H2AC14, H2AC6, H2BC13, H3C13, H4-16]
R-HSA:5693532	DNA Double-Strand Break Repair	REACTOME_Pathways_25.05.2022	1.18E-09	8.24E-08	7.66E-12	1.79E-11	Group 5	5.95	10.00	[BAZ1B, H2BC13, H4-16, LIG3, MDC1, PARP1, RFC1, RIF1, XRCC5, XRCC6]
R-HSA:5693538	Homology Directed Repair	REACTOME_Pathways_25.05.2022	2.02E-05	3.83E-05	7.66E-12	1.79E-11	Group 5	4.35	6.00	[H2BC13, H4-16, LIG3, MDC1, PARP1, RFC1]
R-HSA:5693565	Recruitment and ATM-mediated phosphorylation of repair and signaling proteins at DNA double strand breaks	REACTOME_Pathways_25.05.2022	2.77E-04	3.03E-04	7.66E-12	1.79E-11	Group 5	5.19	4.00	[BAZ1B, H2BC13, H4-16, MDC1]
R-HSA:5693571	Nonhomologous End-Joining (NHEJ)	REACTOME_Pathways_25.05.2022	3.20E-07	1.49E-06	7.66E-12	1.79E-11	Group 5	8.82	6.00	[H2BC13, H4-16, MDC1, RIF1, XRCC5, XRCC6]
R-HSA:5693606	DNA Double Strand Break Response	REACTOME_Pathways_25.05.2022	2.91E-04	3.14E-04	7.66E-12	1.79E-11	Group 5	5.13	4.00	[BAZ1B, H2BC13, H4-16, MDC1]
R-HSA:73927	Depurination	REACTOME_Pathways_25.05.2022	8.01E-05	1.04E-04	7.66E-12	1.79E-11	Group 5	7.14	4.00	[H2AC14, H2AC6, H2BC13, H4-16]
R-HSA:73928	Depyrimidination	REACTOME_Pathways_25.05.2022	1.12E-04	1.31E-04	7.66E-12	1.79E-11	Group 5	6.56	4.00	[H2AC14, H2AC6, H2BC13, H4-16]
R-HSA:73929	Base-Excision Repair, AP Site Formation	REACTOME_Pathways_25.05.2022	1.27E-04	1.46E-04	7.66E-12	1.79E-11	Group 5	6.35	4.00	[H2AC14, H2AC6, H2BC13, H4-16]

S1c

ID	Term	Ontology Source	Term PValue	Term PValue Corrected with Benjamini-Hochberg	Group PValue	Group PValue Corrected with Benjamini-Hochberg	GOLevels	GOGroups	% Associated Genes	Nr. Genes	Associated Genes Found
R-HSA:110328	Recognition and association of DNA glycosylase with site containing an affected pyrimidine	REACTOME_Pathways_25.05.2022	1.12E-04	1.31E-04	2.74E-18	1.92E-17	[-1]	Group 6	6.56	4.00	[H2AC14, H2AC6, H2BC13, H4-16]
R-HSA:110329	Cleavage of the damaged pyrimidine	REACTOME_Pathways_25.05.2022	1.12E-04	1.31E-04	2.74E-18	1.92E-17	[-1]	Group 6	6.56	4.00	[H2AC14, H2AC6, H2BC13, H4-16]
R-HSA:110330	Recognition and association of DNA glycosylase with site containing an affected purine	REACTOME_Pathways_25.05.2022	8.01E-05	1.04E-04	2.74E-18	1.92E-17	[-1]	Group 6	7.14	4.00	[H2AC14, H2AC6, H2BC13, H4-16]
R-HSA:110331	Cleavage of the damaged purine	REACTOME_Pathways_25.05.2022	8.01E-05	1.04E-04	2.74E-18	1.92E-17	[-1]	Group 6	7.14	4.00	[H2AC14, H2AC6, H2BC13, H4-16]
R-HSA:1221632	Meiotic synapsis	REACTOME_Pathways_25.05.2022	2.95E-08	3.44E-07	2.74E-18	1.92E-17	[-1]	Group 6	8.86	7.00	[H2AC14, H2AC6, H2BC13, H4-16, RAD21, SMC3, STAG2]
R-HSA:140342	Apoptosis induced DNA fragmentation	REACTOME_Pathways_25.05.2022	1.21E-09	4.23E-08	2.74E-18	1.92E-17	[-1]	Group 6	38.46	5.00	[H1-0, H1-2, H1-4, HMGB1, HMGB2]
R-HSA:1474165	Reproduction	REACTOME_Pathways_25.05.2022	1.04E-07	6.65E-07	2.74E-18	1.92E-17	[-1]	Group 6	5.59	8.00	[H2AC14, H2AC6, H2BC13, H3C13, H4-16, RAD21, SMC3, STAG2]
R-HSA:1500620	Meiosis	REACTOME_Pathways_25.05.2022	2.32E-08	3.25E-07	2.74E-18	1.92E-17	[-1]	Group 6	6.78	8.00	[H2AC14, H2AC6, H2BC13, H3C13, H4-16, RAD21, SMC3, STAG2]
R-HSA:157579	Telomere Maintenance	REACTOME_Pathways_25.05.2022	6.43E-06	1.50E-05	2.74E-18	1.92E-17	[-1]	Group 6	5.31	6.00	[ATRX, H2AC14, H2AC6, H2BC13, H4-16, RFC1]
R-HSA:171306	Packaging Of Telomere Ends	REACTOME_Pathways_25.05.2022	5.98E-05	8.37E-05	2.74E-18	1.92E-17	[-1]	Group 6	7.69	4.00	[H2AC14, H2AC6, H2BC13, H4-16]
R-HSA:1912408	Pre-NOTCH Transcription and Translation	REACTOME_Pathways_25.05.2022	3.82E-05	5.81E-05	2.74E-18	1.92E-17	[-1]	Group 6	5.38	5.00	[H2AC14, H2AC6, H2BC13, H3C13, H4-16]
R-HSA:1912422	Pre-NOTCH Expression and Processing	REACTOME_Pathways_25.05.2022	8.17E-05	1.04E-04	2.74E-18	1.92E-17	[-1]	Group 6	4.59	5.00	[H2AC14, H2AC6, H2BC13, H3C13, H4-16]
R-HSA:201722	Formation of the beta-catenin:TCF transactivating complex	REACTOME_Pathways_25.05.2022	3.44E-05	5.46E-05	2.74E-18	1.92E-17	[-1]	Group 6	5.49	5.00	[H2AC14, H2AC6, H2BC13, H3C13, H4-16]
R-HSA:212165	Epigenetic regulation of gene expression	REACTOME_Pathways_25.05.2022	2.30E-06	6.44E-06	2.74E-18	1.92E-17	[-1]	Group 6	4.70	7.00	[BAZ1B, CBX3, H2AC14, H2AC6, H2BC13, H3C13, H4-16]
R-HSA:212300	PRC2 methylates histones and DNA	REACTOME_Pathways_25.05.2022	1.17E-05	2.41E-05	2.74E-18	1.92E-17	[-1]	Group 6	6.85	5.00	[H2AC14, H2AC6, H2BC13, H3C13, H4-16]
R-HSA:2299718	Condensation of Prophase Chromosomes	REACTOME_Pathways_25.05.2022	1.25E-05	2.51E-05	2.74E-18	1.92E-17	[-1]	Group 6	6.76	5.00	[H2AC14, H2AC6, H2BC13, H3C13, H4-16]
R-HSA:2468052	Establishment of Sister Chromatid Cohesion	REACTOME_Pathways_25.05.2022	8.24E-08	6.41E-07	2.74E-18	1.92E-17	[-1]	Group 6	36.36	4.00	[PDS5A, RAD21, SMC3, STAG2]
R-HSA:2470946	Cohesin Loading onto Chromatin	REACTOME_Pathways_25.05.2022	5.26E-08	4.60E-07	2.74E-18	1.92E-17	[-1]	Group 6	40.00	4.00	[PDS5A, RAD21, SMC3, STAG2]
R-HSA:2559580	Oxidative Stress Induced Senescence	REACTOME_Pathways_25.05.2022	1.56E-04	1.73E-04	2.74E-18	1.92E-17	[-1]	Group 6	4.00	5.00	[H2AC14, H2AC6, H2BC13, H3C13, H4-16]

S1d Cont.

ID	Term	Ontology Source	Term PValue	Term PValue Corrected with Benjamini-Hochberg	Group PValue	Group PValue Corrected with Benjamini-Hochberg	GOLevels	GOGroups	% Associated Genes	Nr. Genes	Associated Genes Found
R-HSA:2559582	Senescence-Associated Secretory Phenotype (SASP)	REACTOME_Pathways_25.05.2022	9.29E-05	1.12E-04	2.74E-18	1.92E-17	[-1]	Group 6	4.46	5.00	[H2AC14, H2AC6, H2BC13, H3C13, H4-16]
R-HSA:2559583	Cellular Senescence	REACTOME_Pathways_25.05.2022	1.21E-06	4.48E-06	2.74E-18	1.92E-17	[-1]	Group 6	4.06	8.00	[H1-0, H1-2, H1-4, H2AC14, H2AC6, H2BC13, H3C13, H4-16]
R-HSA:2559584	Formation of Senescence-Associated Heterochromatin Foci (SAHF)	REACTOME_Pathways_25.05.2022	3.56E-05	5.54E-05	2.74E-18	1.92E-17	[-1]	Group 6	18.75	3.00	[H1-0, H1-2, H1-4]
R-HSA:2559586	DNA Damage/Telomere Stress Induced Senescence	REACTOME_Pathways_25.05.2022	3.22E-08	3.22E-07	2.74E-18	1.92E-17	[-1]	Group 6	8.75	7.00	[H1-0, H1-2, H1-4, H2AC14, H2AC6, H2BC13, H4-16]
R-HSA:3108214	SUMOylation of DNA damage response and repair proteins	REACTOME_Pathways_25.05.2022	1.52E-05	2.96E-05	2.74E-18	1.92E-17	[-1]	Group 6	6.49	5.00	[MDC1, PARP1, RAD21, SMC3, STAG2]
R-HSA:3214815	HDACs deacetylate histones	REACTOME_Pathways_25.05.2022	4.02E-05	5.98E-05	2.74E-18	1.92E-17	[-1]	Group 6	5.32	5.00	[H2AC14, H2AC6, H2BC13, H3C13, H4-16]
R-HSA:3214847	HATs acetylate histones	REACTOME_Pathways_25.05.2022	2.38E-05	4.27E-05	2.74E-18	1.92E-17	[-1]	Group 6	4.23	6.00	[H2AC14, H2AC6, H2BC13, H3C13, H4-16, HAT1]
R-HSA:3214858	RMTs methylate histone arginines	REACTOME_Pathways_25.05.2022	3.06E-04	3.24E-04	2.74E-18	1.92E-17	[-1]	Group 6	5.06	4.00	[H2AC14, H2AC6, H3C13, H4-16]
R-HSA:427359	SIRT1 negatively regulates rRNA expression	REACTOME_Pathways_25.05.2022	8.26E-06	1.75E-05	2.74E-18	1.92E-17	[-1]	Group 6	7.35	5.00	[H2AC14, H2AC6, H2BC13, H3C13, H4-16]
R-HSA:427389	ERCC6 (CSB) and EHMT2 (G9a) positively regulate rRNA expression	REACTOME_Pathways_25.05.2022	6.23E-07	2.57E-06	2.74E-18	1.92E-17	[-1]	Group 6	7.89	6.00	[CBX3, H2AC14, H2AC6, H2BC13, H3C13, H4-16]
R-HSA:427413	NoRC negatively regulates rRNA expression	REACTOME_Pathways_25.05.2022	7.48E-05	9.87E-05	2.74E-18	1.92E-17	[-1]	Group 6	4.67	5.00	[H2AC14, H2AC6, H2BC13, H3C13, H4-16]
R-HSA:5250913	Positive epigenetic regulation of rRNA expression	REACTOME_Pathways_25.05.2022	2.28E-07	1.14E-06	2.74E-18	1.92E-17	[-1]	Group 6	6.60	7.00	[BAZ1B, CBX3, H2AC14, H2AC6, H2BC13, H3C13, H4-16]
R-HSA:5250924	B-WICH complex positively regulates rRNA expression	REACTOME_Pathways_25.05.2022	1.81E-06	5.77E-06	2.74E-18	1.92E-17	[-1]	Group 6	6.59	6.00	[BAZ1B, H2AC14, H2AC6, H2BC13, H3C13, H4-16]
R-HSA:5250941	Negative epigenetic regulation of rRNA expression	REACTOME_Pathways_25.05.2022	8.53E-05	1.07E-04	2.74E-18	1.92E-17	[-1]	Group 6	4.55	5.00	[H2AC14, H2AC6, H2BC13, H3C13, H4-16]
R-HSA:5334118	DNA methylation	REACTOME_Pathways_25.05.2022	6.61E-06	1.49E-05	2.74E-18	1.92E-17	[-1]	Group 6	7.69	5.00	[H2AC14, H2AC6, H2BC13, H3C13, H4-16]
R-HSA:5578749	Transcriptional regulation by small RNAs	REACTOME_Pathways_25.05.2022	7.15E-05	9.62E-05	2.74E-18	1.92E-17	[-1]	Group 6	4.72	5.00	[H2AC14, H2AC6, H2BC13, H3C13, H4-16]
R-HSA:5617472	Activation of anterior HOX genes in hindbrain development during early embryogenesis	REACTOME_Pathways_25.05.2022	1.34E-04	1.51E-04	2.74E-18	1.92E-17	[-1]	Group 6	4.13	5.00	[H2AC14, H2AC6, H2BC13, H3C13, H4-16]
R-HSA:5619507	Activation of HOX genes during differentiation	REACTOME_Pathways_25.05.2022	1.34E-04	1.51E-04	2.74E-18	1.92E-17	[-1]	Group 6	4.13	5.00	[H2AC14, H2AC6, H2BC13, H3C13, H4-16]
R-HSA:5625740	RHO GTPases activate PKNs	REACTOME_Pathways_25.05.2022	4.23E-05	6.17E-05	2.74E-18	1.92E-17	[-1]	Group 6	5.26	5.00	[H2AC14, H2AC6, H2BC13, H3C13, H4-16]

S1d Cont.

ID	Term	Ontology Source	Term PValue	Term PValue Corrected with Benjamini-Hochberg	Group PValue	Group PValue Corrected with Benjamini-Hochberg	GOLevels	GOGroups	% Associated Genes	Nr. Genes	Associated Genes Found
R-HSA:5625886	Activated PKN1 stimulates transcription of AR (androgen receptor) regulated genes KLK2 and KLK3	REACTOME_Pathways_25.05.2022	7.68E-06	1.68E-05	2.74E-18	1.92E-17	[-1]	Group 6	7.46	5.00	[H2AC14, H2AC6, H2BC13, H3C13, H4-16]
R-HSA:5693532	DNA Double-Strand Break Repair	REACTOME_Pathways_25.05.2022	1.18E-09	8.24E-08	2.74E-18	1.92E-17	[-1]	Group 6	5.95	10.00	[BAZ1B, H2BC13, H4-16, LIG3, MDC1, PARP1, RFC1, RIF1, XRCC5, XRCC6]
R-HSA:5693538	Homology Directed Repair	REACTOME_Pathways_25.05.2022	2.02E-05	3.83E-05	2.74E-18	1.92E-17	[-1]	Group 6	4.35	6.00	[H2BC13, H4-16, LIG3, MDC1, PARP1, RFC1]
R-HSA:5693565	Recruitment and ATM-mediated phosphorylation of repair and signaling proteins at DNA double strand breaks	REACTOME_Pathways_25.05.2022	2.77E-04	3.03E-04	2.74E-18	1.92E-17	[-1]	Group 6	5.19	4.00	[BAZ1B, H2BC13, H4-16, MDC1]
R-HSA:5693606	DNA Double Strand Break Response	REACTOME_Pathways_25.05.2022	2.91E-04	3.14E-04	2.74E-18	1.92E-17	[-1]	Group 6	5.13	4.00	[BAZ1B, H2BC13, H4-16, MDC1]
R-HSA:606279	Deposition of new CENPA-containing nucleosomes at the centromere	REACTOME_Pathways_25.05.2022	1.25E-05	2.51E-05	2.74E-18	1.92E-17	[-1]	Group 6	6.76	5.00	[H2AC14, H2AC6, H2BC13, H4-16, RSF1]
R-HSA:68616	Assembly of the ORC complex at the origin of replication	REACTOME_Pathways_25.05.2022	3.49E-07	1.53E-06	2.74E-18	1.92E-17	[-1]	Group 6	8.70	6.00	[H2AC14, H2AC6, H2BC13, H3C13, H4-16, ORC2]
R-HSA:68867	Assembly of the pre-replicative complex	REACTOME_Pathways_25.05.2022	2.48E-05	4.33E-05	2.74E-18	1.92E-17	[-1]	Group 6	4.20	6.00	[H2AC14, H2AC6, H2BC13, H3C13, H4-16, ORC2]
R-HSA:68875	Mitotic Prophase	REACTOME_Pathways_25.05.2022	1.67E-06	5.55E-06	2.74E-18	1.92E-17	[-1]	Group 6	4.93	7.00	[H2AC14, H2AC6, H2BC13, H3C13, H4-16, NUMA1, TMPO]
R-HSA:68884	Mitotic Telophase/Cytokinesis	REACTOME_Pathways_25.05.2022	1.78E-07	9.56E-07	2.74E-18	1.92E-17	[-1]	Group 6	30.77	4.00	[PDS5A, RAD21, SMC3, STAG2]
R-HSA:73728	RNA Polymerase I Promoter Opening	REACTOME_Pathways_25.05.2022	5.66E-06	1.41E-05	2.74E-18	1.92E-17	[-1]	Group 6	7.94	5.00	[H2AC14, H2AC6, H2BC13, H3C13, H4-16]
R-HSA:73772	RNA Polymerase I Promoter Escape	REACTOME_Pathways_25.05.2022	1.81E-06	5.77E-06	2.74E-18	1.92E-17	[-1]	Group 6	6.59	6.00	[CBX3, H2AC14, H2AC6, H2BC13, H3C13, H4-16]
R-HSA:73854	RNA Polymerase I Promoter Clearance	REACTOME_Pathways_25.05.2022	5.50E-06	1.43E-05	2.74E-18	1.92E-17	[-1]	Group 6	5.45	6.00	[CBX3, H2AC14, H2AC6, H2BC13, H3C13, H4-16]
R-HSA:73864	RNA Polymerase I Transcription	REACTOME_Pathways_25.05.2022	6.10E-06	1.47E-05	2.74E-18	1.92E-17	[-1]	Group 6	5.36	6.00	[CBX3, H2AC14, H2AC6, H2BC13, H3C13, H4-16]
R-HSA:73884	Base Excision Repair	REACTOME_Pathways_25.05.2022	8.56E-08	5.99E-07	2.74E-18	1.92E-17	[-1]	Group 6	7.61	7.00	[H2AC14, H2AC6, H2BC13, H4-16, LIG3, PARP1, RFC1]
R-HSA:73886	Chromosome Maintenance	REACTOME_Pathways_25.05.2022	1.59E-06	5.56E-06	2.74E-18	1.92E-17	[-1]	Group 6	4.96	7.00	[ATRX, H2AC14, H2AC6, H2BC13, H4-16, RFC1, RSF1]
R-HSA:73927	Depurination	REACTOME_Pathways_25.05.2022	8.01E-05	1.04E-04	2.74E-18	1.92E-17	[-1]	Group 6	7.14	4.00	[H2AC14, H2AC6, H2BC13, H4-16]
R-HSA:73928	Depyrimidination	REACTOME_Pathways_25.05.2022	1.12E-04	1.31E-04	2.74E-18	1.92E-17	[-1]	Group 6	6.56	4.00	[H2AC14, H2AC6, H2BC13, H4-16]
R-HSA:73929	Base-Excision Repair, AP Site Formation	REACTOME_Pathways_25.05.2022	1.27E-04	1.46E-04	2.74E-18	1.92E-17	[-1]	Group 6	6.35	4.00	[H2AC14, H2AC6, H2BC13, H4-16]

S1d Cont.

ID	Term	Ontology Source	Term PValue	Term PValue Corrected with Benjamini-Hochberg	Group PValue	Group PValue Corrected with Benjamini-Hochberg	GOLevels	GOGroups	% Associated Genes	Nr. Genes	Associated Genes Found
R-HSA:73933	Resolution of Abasic Sites (AP sites)	REACTOME_Pathways_25.05.2022	5.44E-04	5.69E-04	2.74E-18	1.92E-17	[-1]	Group 6	7.69	3.00	[LIG3, PARP1, RFC1]
R-HSA:75153	Apoptotic execution phase	REACTOME_Pathways_25.05.2022	2.16E-06	6.59E-06	2.74E-18	1.92E-17	[-1]	Group 6	9.62	5.00	[H1-0, H1-2, H1-4, HMGB1, HMGB2]
R-HSA:774815	Nucleosome assembly	REACTOME_Pathways_25.05.2022	1.25E-05	2.51E-05	2.74E-18	1.92E-17	[-1]	Group 6	6.76	5.00	[H2AC14, H2AC6, H2BC13, H4-16, RSF1]
R-HSA:8936459	RUNX1 regulates genes involved in megakaryocyte differentiation and platelet function	REACTOME_Pathways_25.05.2022	4.67E-05	6.68E-05	2.74E-18	1.92E-17	[-1]	Group 6	5.15	5.00	[H2AC14, H2AC6, H2BC13, H3C13, H4-16]
R-HSA:9018519	Estrogen-dependent gene expression	REACTOME_Pathways_25.05.2022	1.51E-07	8.83E-07	2.74E-18	1.92E-17	[-1]	Group 6	5.33	8.00	[H2AC14, H2AC6, H2BC13, H3C13, H4-16, RAD21, SMC3, STAG2]
R-HSA:912446	Meiotic recombination	REACTOME_Pathways_25.05.2022	2.61E-05	4.46E-05	2.74E-18	1.92E-17	[-1]	Group 6	5.81	5.00	[H2AC14, H2AC6, H2BC13, H3C13, H4-16]
R-HSA:9610379	HCMV Late Events	REACTOME_Pathways_25.05.2022	1.10E-04	1.30E-04	2.74E-18	1.92E-17	[-1]	Group 6	4.31	5.00	[H2AC14, H2AC6, H2BC13, H3C13, H4-16]
R-HSA:9616222	Transcriptional regulation of granulopoiesis	REACTOME_Pathways_25.05.2022	3.26E-05	5.30E-05	2.74E-18	1.92E-17	[-1]	Group 6	5.56	5.00	[H2AC14, H2AC6, H2BC13, H3C13, H4-16]
R-HSA:9645723	Diseases of programmed cell death	REACTOME_Pathways_25.05.2022	6.23E-05	8.55E-05	2.74E-18	1.92E-17	[-1]	Group 6	4.85	5.00	[H2AC14, H2AC6, H2BC13, H3C13, H4-16]
R-HSA:9670095	Inhibition of DNA recombination at telomere	REACTOME_Pathways_25.05.2022	8.26E-06	1.75E-05	2.74E-18	1.92E-17	[-1]	Group 6	7.35	5.00	[ATRX, H2AC14, H2AC6, H2BC13, H4-16]
R-HSA:9710421	Defective pyroptosis	REACTOME_Pathways_25.05.2022	1.17E-05	2.41E-05	2.74E-18	1.92E-17	[-1]	Group 6	6.85	5.00	[H2AC14, H2AC6, H2BC13, H3C13, H4-16]
R-HSA:977225	Amyloid fiber formation	REACTOME_Pathways_25.05.2022	8.90E-05	1.09E-04	2.74E-18	1.92E-17	[-1]	Group 6	4.50	5.00	[H2AC14, H2AC6, H2BC13, H3C13, H4-16]

S1d Cont.

Figure S1. Detailed list of the 62 top HMGA2 interaction partners selected based on Reactome pathway algorithm. The seven groups are coded by the same color as they are depicted in Fig. 6a+b.
